# First-principles study of superconducting hydrogen sulfide at pressure up to 500 GPa

**DOI:** 10.1038/s41598-017-04714-5

**Published:** 2017-06-30

**Authors:** Artur P. Durajski, Radosław Szczęśniak

**Affiliations:** 1 Institute of Physics, Częstochowa University of Technology, Ave. Armii Krajowej 19, 42-200 Częstochowa, Poland; 2 Institute of Physics, Jan Długosz University in Częstochowa, Ave. Armii Krajowej 13/15, 42-200 Częstochowa, Poland

## Abstract

We investigate the possibility of achieving the room-temperature superconductivity in hydrogen sulfide (H_3_S) through increasing external pressure, a path previously widely used to reach metallization and superconducting state in novel hydrogen-rich materials. The electronic properties and superconductivity of H_3_S in the pressure range of 250–500 GPa are determined by the first-principles calculations. The metallic character of a body-centered cubic Im$\overline{{\bf{3}}}$m structure is found over the whole studied pressure. Moreover, the absence of imaginary frequency in phonon spectrum implies that this structure is dynamically stable. Furthermore, our calculations conducted within the framework of the Eliashberg formalism indicate that H_3_S in the range of the extremely high pressures is a conventional strong-coupling superconductor with a high superconducting critical temperature, however, the maximum critical temperature does not exceed the value of 203 K.

## Introduction

The recent breakthrough theoretical calculations^1, 2^ confirmed by spectacular experimental reports of the superconductivity in hydrogen sulfide, with the record high critical temperature equals to 203 K at pressure closed to 150 GPa^3–5^, open the door to achieving the room-temperature superconductivity in the compressed hydrogen-rich materials^6–8^ or in the pristine metallic hydrogen^9–12^. In contrast to the cuprates^13, 14^, where the mechanism responsible for the superconducting state is still debated^15–22^, the phonon-mediated pairing scenario is generally accepted in the case of H_3_S^23–27^ due to the observed of a large isotope effect which convincingly suggest that hydrogen sulfide is a conventional superconductor^3, 4^. Therefore, in the theoretical papers^28–32^, the superconducting properties of hydrogen sulfide are studied in the framework of the mean-field Bardeen-Cooper-Schrieffer (BCS) theory^33, 34^, or more precisely using the Migdal-Eliashberg (ME) approach^35–37^. For the crystal structures of high-T_*C*_ hydrides, the high-pressure x-ray diffraction experiments combined with the electrical resistance measurements^5, 38^ confirm that H_3_S takes a body-centered cubic $Im\overline{3}m$ structure, which is certainly stable above 180 GPa^2, 39^. The pressure-temperature phase diagram of solid hydrogen sulfide, determined on the base of a sharp falls to zero in resistivity with cooling, shows increase of *T*_*C*_ from 95 to 203 K in the range of pressure from 110 to 155 GPa^3, 5^ and, then, further increasing of compression causes a linear decrease of critical temperature to 170 K at 225 GPa^3^. There arises the natural question, whether under the influence of the high pressure is it possible to obtain the superconducting state with the value of the critical temperature even higher than 203 K in the case of H_3_S compound. Below, we present the results of the *ab initio* calculations, which conclude that *T*_*C*_ does not exceed the value of 203 K, while the range of the pressure from 250 to 500 GPa is adopted.

## Computational details

**Figure 1:**
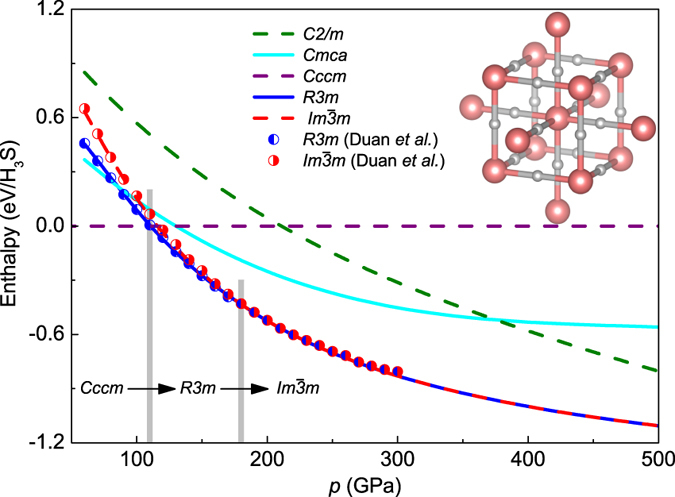
The influence of the pressure on the value of the enthalpy for selected crystal structures. The symbols represent the results obtained by Duan *et al*.^2^. Moreover, the $Im\overline{3}m$ crystal structure assumed for H_3_S is included.

**Figure 2:**
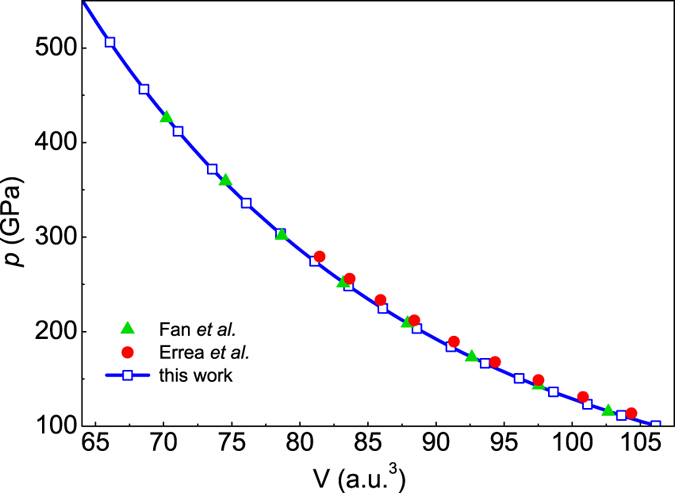
The calculated volume-pressure data in the classical nuclei limit (the equation of states). The filled symbols are related to the results presented in publications^39, 56^.

The searches for the stable high pressure structures of H_3_S system were performed through the evolutionary algorithm implemented in the USPEX code^40, 41^, which has been applied successfully to a number of compressed systems containing hydrogen^42, 43^. The computed enthalpy differences relative to the *Cccm* structure (*H* − *H*_*Cccm*_) as a function of pressure for the selected crystal structures are presented in Fig. 1. It can be clearly seen that at low pressure (below 110 GPa) the lowest value of enthalpy corresponds to the orthorhombic *Cccm* structure and the hexagonal *R*3*m* structure has the most stable lattice between 110 and 180 GPa. Then, a cubic $Im\overline{3}m$ structure becomes favorable above 180 GPa. This structure is characterized by two S atoms located at a simple body centered cubic lattice and H atom situated midway between the two S atoms (see the inset in Fig. 1). Let us emphasize that for the low pressures our results agree well with the data reported by Duan *et al*.^2^. Moreover, for H_3_S the second-order structural phase transition from *R*3*m* to $Im\overline{3}m$ is also experimentally observed but for a slightly lower pressure (~150 GPa)^5, 44^. It should be underlined that over 450 various structures were studied, wherein in any case was obtained enthalpy lower than ${H}_{Im\overline{3}m}$ in the range of pressures from 250 to 500 GPa. Due to the above fact in this study the critical temperature and the other thermodynamic parameters of the superconducting state of H_3_S are calculated only for the structure $Im\overline{3}m$. Figure 2 presents the curve of the volume-pressure type. This curve can be reproduced with the help of the third-order Birch-Murnaghan equation: $p(V)=\frac{3}{2}{B}_{0}[{({V}_{0}/V)}^{\mathrm{7/3}}-{({V}_{0}/V)}^{\mathrm{5/3}}]\,\{1+\frac{3}{4}({B}_{0}^{^{\prime} }-4)[{({V}_{0}/V)}^{\mathrm{2/3}}-1]\}$, where *B*_0_ = 129.8 GPa, ${V}_{0}=158.4\,{{\rm{a}}}_{0}^{3}$, and ${B}_{0}^{^{\prime} }=3.6$ ^39^.

The characteristics of the electron structure, the phonon structure, and the electron-phonon interaction was made in the framework of the Quantum-ESPRESSO package^45^. The calculations were conducted basing on the density-functional methods using the PWSCF code^45–47^. The Vanderbilt-type ultra-soft pseudopotentials for S and H atoms were employed with the kinetic energy cut-off equal to 80 Ry. The phonon calculations were performed for 32 × 32 × 32 Monkhorst-Pack *k*-mesh with the Gaussian smearing of 0.03 Ry. The electron-phonon coupling matrices were computed using 8 × 8 × 8 **q**-grid. The superconducting transition temperature (*T*_*C*_) can be in a simple way estimated using the Allen-Dynes modified McMillan equation^48^:

1 $${k}_{B}{T}_{C}={f}_{1}{f}_{2}\frac{{\omega }_{\mathrm{ln}}}{1.2}\,\exp \,[\frac{-1.04(1+\lambda )}{\lambda -{\mu }^{\ast }(1+0.62\lambda )}],$$

where *k*_*B*_ = 0.0862 meV/K (the Boltzmann constant), *f*_1_ and *f*_2_ are the correction functions:

2 $${f}_{1}={[1+{(\frac{\lambda }{2.46(1+3.8{\mu }^{\ast })})}^{\mathrm{3/2}}]}^{\mathrm{1/3}},$$

3 $${f}_{2}=1+\frac{(\sqrt{{\omega }_{2}}/{\omega }_{\mathrm{ln}}-1){\lambda }^{2}}{{\lambda }^{2}+{[1.82(1+6.3{\mu }^{\ast })(\sqrt{{\omega }_{2}}/{\omega }_{\mathrm{ln}})]}^{2}}.$$

The quantity *ω*_2_ represents the second moment of the normalized weight function:

4 $${\omega }_{2}\equiv \frac{2}{\lambda }{\int }_{0}^{{\omega }_{D}}\,d\omega {\alpha }^{2}F(\omega )\omega $$

and *ω*_ln_ is the logarithmic average of the phonon frequencies:

5 $${\omega }_{\mathrm{ln}}\equiv \exp \,[\frac{2}{\lambda }{\int }_{0}^{{\omega }_{D}}\,d\omega \frac{{\alpha }^{2}F(\omega )}{\omega }\,\mathrm{ln}(\omega )].$$

More sophisticated calculations can be conducted within the framework of the Eliashberg formalism, which allows a more accurately description of the superconducting state in strong-coupling systems^31, 32^. The Eliashberg equations for the superconducting order parameter function ${\phi }_{m}\equiv \phi \,(i{\omega }_{m})$ and the electron mass renormalization function ${Z}_{m}\equiv Z\,(i{\omega }_{m})$ written in the imaginary-axis formulation take the following form refs 36 and 49:

6 $${\phi }_{m}=\pi {k}_{B}T\sum _{n=-M}^{M}\,\frac{{\lambda }_{n,m}-{\mu }^{\ast }\theta ({\omega }_{c}-|{\omega }_{n}|)}{\sqrt{{\omega }_{n}^{2}{Z}_{n}^{2}+{\phi }_{n}^{2}}}{\phi }_{n},$$

and

7 $${Z}_{m}=1+\frac{\pi {k}_{B}T}{{\omega }_{n}}\sum _{n=-M}^{M}\,\frac{{\lambda }_{n,m}}{\sqrt{{\omega }_{n}^{2}{Z}_{n}^{2}+{\phi }_{n}^{2}}}{\omega }_{n}{Z}_{n},$$

where the pairing kernel for the electron-phonon interaction is given by:

8 $${\lambda }_{n,m}=2{\int }_{0}^{{\omega }_{D}}\,d\omega \frac{\omega }{{({\omega }_{n}-{\omega }_{m})}^{2}+{\omega }^{2}}{\alpha }^{2}F(\omega ).$$

Symbols ${\mu }^{\ast }$ and *θ* denote the Coulomb pseudopotential and the Heaviside function with cut-off frequency *ω*_*c*_ equal to three times the maximum phonon frequency (*ω*_*D*_). The *α* ^2^ *F*(*ω*) functions, called the Eliashberg functions, for H_3_S system were calculated using the density functional perturbation theory and the plane-wave pseudopotential method, as implemented in the Quantum-Espresso package^45^:

9 $${\alpha }^{2}F(\omega )=\frac{1}{2\pi \rho ({\varepsilon }_{F})}\sum _{{\bf{q}}\nu }\,\delta (\omega -{\omega }_{{\bf{q}}\nu })\frac{{\gamma }_{{\bf{q}}\nu }}{{\omega }_{{\bf{q}}\nu }},$$

with

10 $${\gamma }_{{\bf{q}}\nu }=2\pi {\omega }_{{\bf{q}}\nu }\sum _{ij}\,\int \frac{{d}^{3}k}{{{\rm{\Omega }}}_{BZ}}{|{g}_{{\bf{q}}\nu }({\bf{k}},i,j)|}^{2}\delta ({\varepsilon }_{{\bf{q}},i}-{\varepsilon }_{F})\delta ({\varepsilon }_{{\bf{k}}+{\bf{q}},j}-{\varepsilon }_{F}),$$

where $\rho \,({\varepsilon }_{F})$ denotes the density of states at the Fermi energy, *ω*_**q***ν*_ determines the values of the phonon energies, and *γ*_**q***ν*_ represents the phonon linewidth. The electron-phonon coefficients are given by *g*_**q***ν*_ (**k**, *i*, *j*) and ${\varepsilon }_{{\bf{k}},i}$ is the electron band energy.

## Results and Discussion

**Figure 3:**
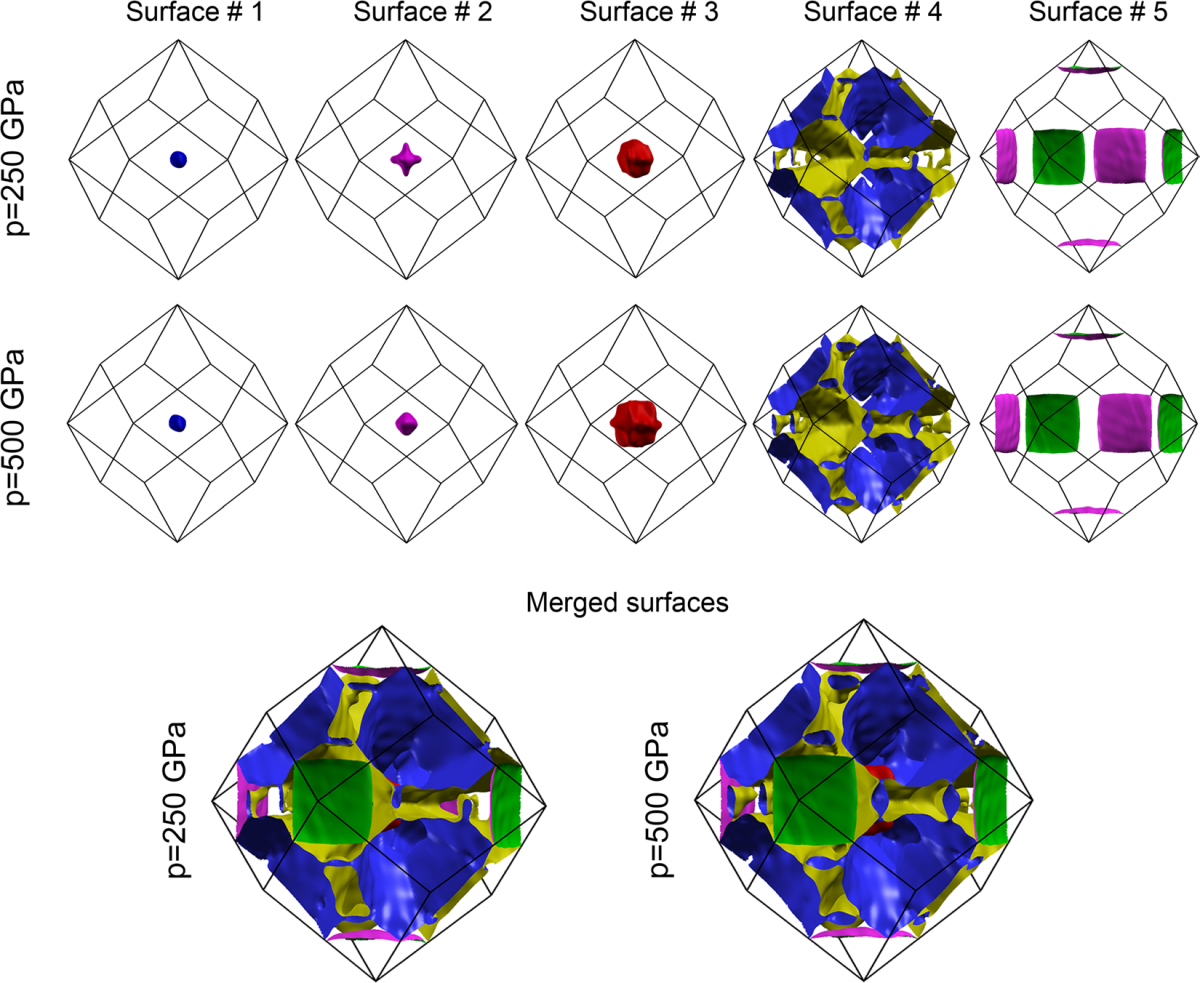
The Fermi surface of the $Im\overline{3}m$ structure of H_3_S at 250 and 500 GPa. (Top) The Fermi surface is formed by 5 different surfaces crossing the Fermi energy. (Bottom) The view of the merged Fermi surfaces at 250 and 500 GPa including all the surfaces.

To investigate the electronic properties of H_3_S at $p\in \langle 250,500\rangle \,{\rm{GPa}}$, we calculate the electronic band structure and density of states (DOS). The Fermi surface of H_3_S at 250 and 500 GPa is shown in Fig. 3. It is formed by five different Fermi surfaces calculated in the bcc Brillouin zone^50^. As has been previously reported by Bianconi and Jarlborg, the red small Fermi surface centered at the Γ-point and covering the surfaces #1 and #2, appears above 95 GPa with the change of the Fermi surface topology. This change of the Fermi surface topology is called a *L1 Lifshitz transition for a new appearing Fermi surface spot* and occurs where the bands at the Γ-point cross the chemical potential. The *L2 Lifshitz transition for neck disrupting* occurs around 180–200 GPa and is connected with appearing of the small tubular necks in the Fermi surface (in particular in the surface #4)^50, 51^.

**Figure 4:**
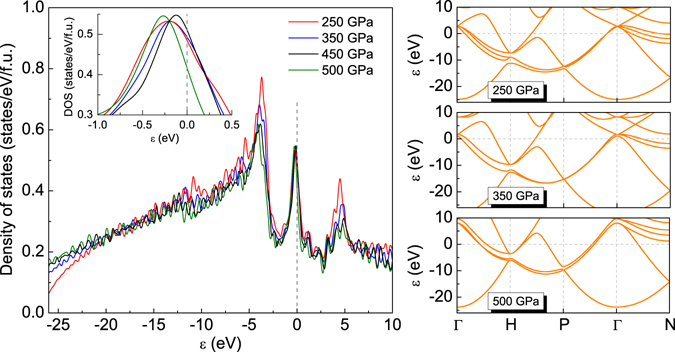
(Left panel) The electron density of states for the selected values of pressure. The insert presents the course of the analyzed function in the vicinity of the Fermi energy. (Right panel) The electron band energy for the selected values of pressure.

The results presented in Fig. 4 clearly show that $Im\overline{3}m$ structure is a good metal with a large DOS at the Fermi level (0.418–0.511 states/eV/f.u.). This is in a good agreement with the previous theoretical and experimental results obtained for the lower pressure^3–5, 52^. The metallic behavior of this system indicates that $Im\overline{3}m$ phase might be superconducting above 250 GPa.

**Figure 5:**
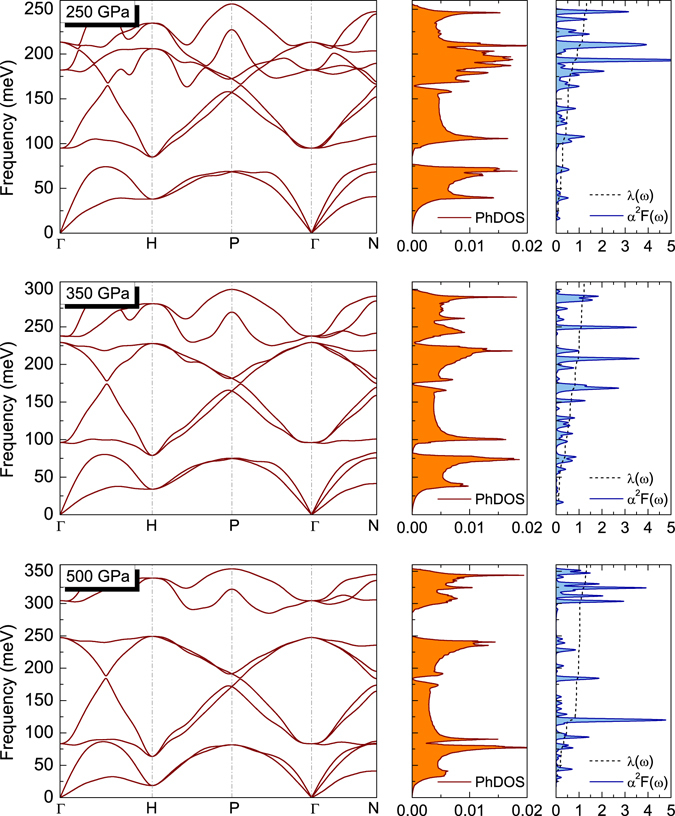
The phonon dispersion relation, the phonon density of states, and the spectral functions for selected values of pressure.

In order to investigate the superconductivity of H_3_S, the phonon band structures, the phonon density of states (PhDOS) and the Eliashberg spectral functions together with the electron-phonon integrals $\lambda (\omega )=2{\int }_{0}^{\omega }\,d\omega {\alpha }^{2}F(\omega )/\omega$ were carried out. As shown in Fig. 5 there is no imaginary frequency to be found in the whole Brillouin zone, confirming that $Im\overline{3}m$ is a dynamically stable structure. In the case of the pressure of 250 GPa, the clearly separated lines respectively associated with the low-energy vibrations of sulfur ($\omega \in \langle \mathrm{0,76.4}\rangle \,{\rm{meV}}$) and the high-energy vibrations of hydrogen ($\omega \in \langle \mathrm{95.2,256.7}\rangle \,{\rm{meV}}$) can be noticed in the phonon dispersive relation. Such fact directly translates into the shape of the function of the phonon density of states, which consists of two parts separated by the gap of the energy (about 19 meV). On the basis of the diagram related to the spectral function it can be seen that the contribution to the electron-phonon coupling constant comes mainly from hydrogen, and is equal approximately to 79%. At the pressure of 350 GPa, the maximum energy of the hydrogen vibrations becomes larger and equals to 308.7 meV. Still visible is the division of functions of the phonon density of states on the part related to sulfur and hydrogen. However, the energy gap width decreases, and is approximately 14 meV. The contribution of hydrogen to the electron-phonon coupling constant is still dominating (around 66%). The increase of the pressure by the further 150 GPa causes the renewed increase in the maximum vibration energy of hydrogen ${[{\omega }_{D}]}_{500{\rm{GPa}}}/{[{\omega }_{D}]}_{350{\rm{GPa}}}=1.179$. However, the division of the phonon density of states on the portion derived from sulfur and hydrogen is galling. The disappearance of the sulfur-hydrogen separation results also in the slight increase of the value of the electron-phonon coupling constant in the range of the lower frequencies ${\lambda }_{500{\rm{GPa}}}(\omega =120\,{\rm{meV}})/{\lambda }_{350{\rm{GPa}}}(\omega =120\,{\rm{meV}})=1.29$. Above the pressure of 500 GPa we found the imaginary (negative) phonon frequencies which is an indication of the structural instability. This is one of the reason why we have limited our calculations to this range of pressures, the second one is that higher pressures are far beyond the ability of the experiment. In light of the latest results on the metallization of hydrogen^53^, compression up to 500 GPa is possible to achieve in laboratory.

**Figure 6:**
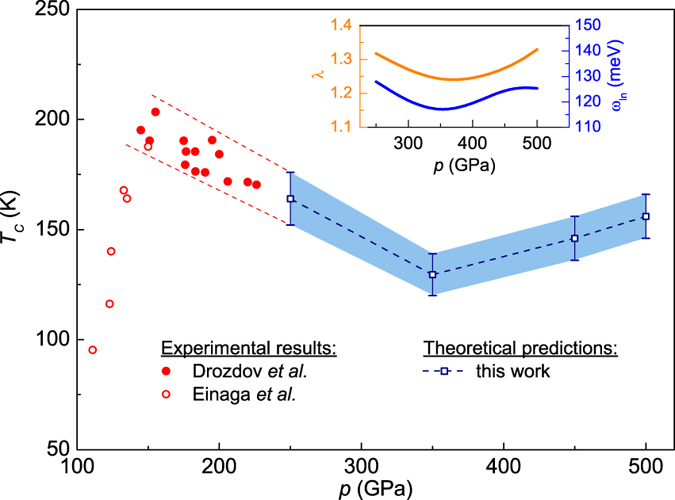
The critical temperature as a function of the pressure. The theoretical results were obtained in the framework of the Eliashberg formalism with commonly accepted value of the Coulomb pseudopotential, ${\mu }^{\ast }=0.13$. The error bars indicate the value range of *T*_*C*_ with ${\mu }^{\ast }\in \langle 0.11,0.15\rangle$. Additionally, the experimental data presented in papers^3, 5^ was presented. The insertion shows the dependence of the electron-phonon coupling constant and the logarithmic phonon frequency on the pressure.

Figure 6 ilustrates the pressure dependence of the superconducting critical temperature. Close and open circles corresponds to the experimental results presented by Drozdov *et al*.^3^ and Einaga *et al*.^5^, respectively. The red dashed lines drawn by eye represents the trend of the experimental data above 150 GPa and great combine together with the theoretical range of *T*_*C*_ calculated for the high pressures. These theoretical results were obtained using the Eliashberg equations and the following relation: ${{\rm{\Delta }}}_{m=1}(T={T}_{C})=0$, where the order parameter is defined as ${{\rm{\Delta }}}_{m=1}={\phi }_{m=1}/{Z}_{m=1}$. The commonly accepted value of the Coulomb pseudopotential ${\mu }^{\ast }=0.13$ was adopted, the exact results of *T*_*C*_ are collected in Table 1. The error bars indicate the value range of *T*_*C*_ with ${\mu }^{\ast }\in \langle 0.11,0.15\rangle$. The obtained results show that *T*_*C*_ decreases from 164 to 129 K in the range of pressure from 250 to 350 GPa. Then above 350 GPa the superconducting critical temperature starts increasing. This is a promising result, however in the range of the pressure from 350 to 500 GPa the critical temperature does not exceed the value of 203 K. This can be explained by the unfavorable, and simultaneously weak, variation of the electron-phonon coupling constant and the logarithmic phonon frequency (the insert in Fig. 6). From the conducted *ab initio* calculations comes the conclusion that this is caused by the small or unfavorable influence of the pressure on the electron density of states and the electron-phonon matrix elements. It can be, however, noticed that the value of *T*_*C*_ in the range of the very high pressures is relatively high and does not drop below 120 K. We did not study the critical temperature under extreme pressures because beyond 500 GPa the H_3_S structure loses the dynamical stability.

**Table 1:** Density of states at the Fermi level *ρ* (*ε*_*F*_) (in units states/eV/f.u.), Fermi energy *ε*_*F*_, electron-phonon coupling constant *λ*, logarithmic phonon frequency *ω*_*ln*_, critical temperature *T*_*C*_ (determined using Eliashberg equations for ${\mu }^{\ast }=0.13$), superconducting energy gap Δ(0) and dimensionless ratio 2Δ(0)/*k*_*B*_ *T*_*C*_ of H_3_S under different pressures.

*p* (GPa)	*a* (Å)	*ρ* (*ε*_*F*_)	*ε*_*F*_ (eV)	*λ*	*ω*_*ln*_ (meV)	*T*_*C*_ (K)	Δ(0) (meV)	2Δ(0)/*k*_*B*_ *T*_*C*_
250	2.913	0.486	19.07	1.31	127.93	164	29.1	4.12
350	2.812	0.497	21.54	1.22	112.13	129	22.5	4.05
450	2.771	0.511	23.65	1.26	126.17	146	25.9	4.12
500	2.699	0.418	24.63	1.32	125.32	156	28.0	4.16

The lattice constant *a* corresponding with appropriate pressure is also included.

Then, by using the analytical continuation^37, 49^, we determine the superconducting energy gap Δ(0) and the dimensionless ratio 2Δ(0)/*T*_*C*_ which in the BCS theory takes the constant value 3.53. As we can see in Table 1, the obtained results significantly exceed the value of BCS predictions. This is connected with the strong-coupling and retardation effects, which in the framework of the Eliashberg formalism are not neglected. During the preparation of this manuscript, a superconducting energy gap of H_3_S compressed to 150 GPa was experimentally found (2Δ = 73 meV)^54^. Taking into account this result and the previously determined value of *T*_*C*_ at this same pressure (203 K)^3^ we can evidence that 2Δ/*k*_*B*_ *T*_*C*_ = 4.17 is surprisingly close to our predictions for higher pressures. The above fact proves the correctness of our calculations.

## Conclusions

In this paper we showed that H_3_S exhibits the superconducting properties in the range of the very high pressures (250–500 GPa), however, the critical temperature does not exceed the value of 203 K. The obtained result is related to the weak and unfavorable volatility of the electron-phonon coupling constant and the logarithmic phonon frequency. From the microscopic point of view, this results from the small or unfavorable influence of the pressure on the value of the electron density of states at the Fermi surface and the electron-phonon matrix elements. According to the above, it can be seen that the increase of the pressure alone is not sufficient to obtain the superconducting state in H_3_S at the room temperature. It is possible that the better method to achieve this goal is the appropriate partial atomic substitution of S atoms by other atoms. Interesting theoretical results were obtained by Ge *et al*. in the paper^55^, where the increase of the P-substitution rate causes the increase of the DOS, the phonon linewidths and the electron-phonon coupling constant. Finally, *T*_*C*_ = 280 K for H_3_S_0.925_P_0.075_ at 250 GPa.

## References

[1] Li Y, Hao J, Liu H, Li Y, Ma Y (2014). The metallization and superconductivity of dense hydrogen sulfide. J. Chem. Phys..

[2] Duan D (2014). Pressure-induced metallization of dense (H_2_S)_2_H_2_ with high-T_*C*_ superconductivity. Sci. Rep..

[3] Drozdov AP, Eremets MI, Troyan IA, Ksenofontov V, Shylin SI (2015). Conventional superconductivity at 203 kelvin at high pressures in the sulfur hydride system. Nature.

[4] Troyan I (2016). Observation of superconductivity in hydrogen sulfide from nuclear resonant scattering. Science.

[5] Einaga M (2016). Crystal structure of the superconducting phase of sulfur hydride. Nat. Phys..

[6] Ashcroft NW (2004). Hydrogen dominant metallic alloys: high temperature superconductors?. Phys. Rev. Lett..

[7] Wang H, Tse JS, Tanaka K, Iitaka T, Ma Y (2012). Superconductive sodalite-like clathrate calcium hydride at high pressures. Proc. Natl. Acad. Sci. USA.

[8] Feng X, Zhang J, Gao G, Liu H, Wang H (2015). Compressed sodalite-like MgH_6_ as a potential high-temperature superconductor. RSC Adv..

[9] Ashcroft NW (1968). Metallic hydrogen: a high-temperature superconductor?. Phys. Rev. Lett..

[10] Cudazzo P (2008). Ab initio description of high-temperature superconductivity in dense molecular hydrogen. Phys. Rev. Lett..

[11] McMahon JM, Ceperley DM (2011). Ground-state structures of atomic metallic hydrogen. Phys. Rev. Lett..

[12] McMahon JM, Ceperley DM (2011). High-temperature superconductivity in atomic metallic hydrogen. Phys. Rev. B.

[13] Bednorz JG, Müller KA (1986). Possible high T_*C*_ superconductivity in the Ba-La-Cu-O system. Z. Phys. B.

[14] Bednorz JG, Müller KA (1988). Perovskite-type oxides - the new approach to high-T_*C*_ superconductivity. Rev. Mod. Phys..

[15] Emery VJ (1987). Theory of high-T_C_ superconductivity in oxides. Phys. Rev. Lett..

[16] Dagotto E (1994). Correlated electrons in high-temperature superconductors. Rev. Mod. Phys..

[17] Damascelli A, Hussain Z, Shen ZX (2003). Angle-resolved photoemission studies of the cuprate superconductors. Rev. Mod. Phys..

[18] Krzyzosiak M, Gonczarek R, Gonczarek A, Jacak L (2016). Applications of the conformal transformation method in studies of composed superconducting systems. Front. Phys..

[19] Cuk T (2005). A review of electron-phonon coupling seen in the high-T_C_ superconductors by angle-resolved photoemission studies (ARPES). Phys. Status Solidi B.

[20] Tarasewicz P, Baran D (2006). Extension of the Fröhlich method to 4-fermion interactions. Phys. Rev. B.

[21] Szczęśniak R (2012). Pairing mechanism for the high-T_*C*_ superconductivity: symmetries and thermodynamic properties. PloS One.

[22] Szczęśniak R, Durajski AP (2014). Anisotropy of the gap parameter in the hole-doped cuprates. Supercond. Sci. Technol..

[23] Mazin II (2016). Superconductivity: Extraordinarily conventional. Nature.

[24] Bernstein N, Hellberg CS, Johannes MD, Mazin II, Mehl MJ (2015). What superconducts in sulfur hydrides under pressure and why. Phys. Rev. B.

[25] Ortenzi L, Cappelluti E, Pietronero L (2016). Band structure and electron-phonon coupling in H_3_S: A tight-binding model. Phys. Rev. B.

[26] Sano W, Koretsune T, Tadano T, Akashi R, Arita R (2016). Effect of van Hove singularities on high-T_*C*_ superconductivity in H_3_S. Phys. Rev. B.

[27] Gor’kov LP, Kresin VZ (2016). Pressure and high-T_*C*_ superconductivity in sulfur hydrides. Sci. Rep..

[28] Errea I (2015). High-pressure hydrogen sulfide from first principles: A strongly anharmonic phonon-mediated superconductor. Phys. Rev. Lett..

[29] Durajski AP, Szczęśniak R, Li Y (2015). Non-BCS thermodynamic properties of H_2_S superconductor. Physica C.

[30] Flores-Livas AJ, Sanna A, Gross EKU (2016). High temperature superconductivity in sulfur and selenium hydrides at high pressure. Eur. Phys. J. B.

[31] Durajski AP, Szczęśniak R, Pietronero L (2016). High-temperature study of superconducting hydrogen and deuterium sulfide. Ann. Phys. (Berlin).

[32] Durajski A (2016). Quantitative analysis of nonadiabatic effects in dense H_3_S and PH_3_ superconductors. Sci. Rep..

[33] Bardeen J, Cooper LN, Schrieffer JR (1957). Microscopic theory of superconductivity. Phys. Rev..

[34] Bardeen J, Cooper LN, Schrieffer JR (1957). Theory of superconductivity. Phys. Rev..

[35] Migdal AB (1958). Interaction between electrons and lattice vibrations in a normal metal. Soviet Physics JETP.

[36] Eliashberg GM (1960). Interactions between electrons and lattice vibrations in a superconductor. Soviet Physics JETP.

[37] Carbotte JP (1990). Properties of boson-exchange superconductors. Rev. Mod. Phys..

[38] Li Y (2016). Dissociation products and structures of solid H_2_S at strong compression. Phys. Rev. B.

[39] Errea I (2016). Quantum hydrogen-bond symmetrization in the superconducting hydrogen sulfide system. Nature.

[40] Oganov AR, Glass CW (2006). Crystal structure prediction using ab initio evolutionary techniques: Principles and applications. J. Chem. Phys Physics.

[41] Glass CW, Oganov AR, Hansen N (2006). USPEX-evolutionary crystal structure prediction. Comput. Phys. Commun..

[42] Esfahani MMD (2016). Superconductivity of novel tin hydrides (Sn_*n*_H_*m*_) under pressure. Sci. Rep..

[43] Liu YX (2015). Structures and properties of osmium hydrides under pressure from first principle calculation. J. Phys. Chem. C.

[44] Einaga M (2017). Two-year progress in experimental investigation on high-temperature superconductivity of sulfur hydride. Jpn. J. Appl. Phys..

[45] Giannozzi P (2009). QUANTUM ESPRESSO: a modular and open-source software project for quantum simulations of materials. J. Phys. Condens. Matter.

[46] Hohenberg P, Kohn W (1964). Inhomogeneous electron gas. Phys. Rev..

[47] Kohn W, Sham LJ (1965). Self-consistent equations including exchange and correlation effects. Phys. Rev..

[48] Allen PB, Dynes RC (1975). Transition temperature of strong-coupled superconductors reanalyzed. Phys. Rev. B.

[49] Marsiglio F, Schossmann M, Carbotte JP (1988). Iterative analytic continuation of the electron self-energy to the real axis. Phys. Rev. B.

[50] Bianconi A, Jarlborg T (2015). Superconductivity above the lowest earth temperature in pressurized sulfur hydride. Europhys. Lett..

[51] Jarlborg T, Bianconi A (2016). Breakdown of the migdal approximation at lifshitz transitions with giant zero-point motion in H_3_S superconductor. Sci. Rep..

[52] Akashi R, Kawamura M, Tsuneyuki S, Nomura Y, Arita R (2015). First-principles study of the pressure and crystal-structure dependences of the superconducting transition temperature in compressed sulfur hydrides. Phys. Rev. B.

[53] Dias, R. P. & Silvera, I. F. Observation of the wigner-huntington transition to metallic hydrogen. *Science* **355**, 715–718 (2017). 10.1126/science.aal1579 28126728

[54] Capitani, F. *et al*. Spectroscopy of H_3_S: evidence of a new energy scale for superconductivity. *arXiv*:*1612*.*06732v2* (2016).

[55] Ge Y, Zhang F, Yao Y (2016). First-principles demonstration of superconductivity at 280 K in hydrogen sulfide with low phosphorus substitution. Phys. Rev. B.

[56] Fan F, Papaconstantopoulos DA, Mehl MJ, Klein BM (2016). Theory of the superconducting state. I. the ground state at the absolute zero of temperature. J. Phys. Chem. Solids.

